# Hybrid Sequencing Approach Applied to Human Fecal Metagenomic Clone Libraries Revealed Clones with Potential Biotechnological Applications

**DOI:** 10.1371/journal.pone.0047654

**Published:** 2012-10-17

**Authors:** Mária Džunková, Giuseppe D’Auria, David Pérez-Villarroya, Andrés Moya

**Affiliations:** 1 Joint Unit of Research in Genomics and Health, Centre for Public Health Research (CSISP) - Cavanilles Institute for Biodiversity and Evolutionary Biology, University of Valencia, Valencia, Spain; 2 CIBER en Epidemiología y Salud Pública (CIBEResp), Madrid, Spain; J. Craig Venter Institute, United States of America

## Abstract

Natural environments represent an incredible source of microbial genetic diversity. Discovery of novel biomolecules involves biotechnological methods that often require the design and implementation of biochemical assays to screen clone libraries. However, when an assay is applied to thousands of clones, one may eventually end up with very few positive clones which, in most of the cases, have to be “domesticated” for downstream characterization and application, and this makes screening both laborious and expensive. The negative clones, which are not considered by the selected assay, may also have biotechnological potential; however, unfortunately they would remain unexplored. Knowledge of the clone sequences provides important clues about potential biotechnological application of the clones in the library; however, the sequencing of clones one-by-one would be very time-consuming and expensive. In this study, we characterized the first metagenomic clone library from the feces of a healthy human volunteer, using a method based on 454 pyrosequencing coupled with a clone-by-clone Sanger end-sequencing. Instead of whole individual clone sequencing, we sequenced 358 clones in a pool. The medium-large insert (7–15 kb) cloning strategy allowed us to assemble these clones correctly, and to assign the clone ends to maintain the link between the position of a living clone in the library and the annotated contig from the 454 assembly. Finally, we found several open reading frames (ORFs) with previously described potential medical application. The proposed approach allows planning *ad-hoc* biochemical assays for the clones of interest, and the appropriate sub-cloning strategy for gene expression in suitable vectors/hosts.

## Introduction

Since the late nineties, metagenomic-based methodologies have been applied to decipher the composition and gene content of bacterial communities in the environment, as well as to detect novel biomolecules for subsequent functional screening [1], [2]. The majority of anti-microbial or anti-cancer drugs have a natural origin [3] and the most disparate environments (sea water, extreme environments, soil) have been studied by metagenomic approaches [4]–[6]. Such studies have led to the discovery of many novel biocatalysts such as lipases or esterases [7]–[10], cellulases [11], [12], chitinases [13], DNA polymerases [14], proteases [15], and a wide range of antibiotics [16]. Symbiotic metagenomes are also promising when it comes to seeking molecules with medical applications given their connection to the healthy status of hosts [17]. Despite this fact, to date no metagenomic library from human symbiotic ecosystems has been screened for novel biomolecules.

Several strategies can be followed to discover new bacterial products in metagenomic samples [18]. One of them is the construction of clone libraries. Small-insert libraries (plasmid vectors) are employed to identify bioproducts encoded by a single gene or a small operon. Large-insert libraries (cosmid vectors or bacterial artificial chromosomes) can be used to isolate larger gene clusters, which could encode for complete pathways [1], [4]. One of the possible screening methods is functional-based screening, in which a given metagenomic library is tested against a wide spectra of screening assays, aiming to identify clones possessing interesting features. Sequence-based screening involves the selection of positive clones for a PCR reaction specifically designed for a gene of interest. A third possible method is substrate-induced gene-expression screening (SIGEX), which has been successfully applied to select clones whose expression is induced by a given substrate [19]. However, in spite of the efforts to screen for natural bioproducts, both discovery rate and application have dramatically declined [20].

In all the above-mentioned methods, an assay designed to discover a single novel biomolecule must be applied to the whole clone library (often containing thousands of clones), finally giving only a few positive clones (if any), which makes the screening process very inefficient. However, the clones that are negative for that specific assay could also contain sequences with an interesting biotechnological potential; however, unfortunately in this case they would remain unexplored.

Prior knowledge of clone sequences can help researchers to design the appropriate screening assays and, thus, to increase the biotechnological application potential of the library (novel biomolecule per clone library rate). However, sequencing clones one-by-one using any type of sequencing platform would be very time-consuming and expensive.

Here, we propose a hybrid sequencing approach based on 454 pyrosequencing coupled with a clone-by-clone Sanger end-sequencing. This technique allows genetic information to be gathered from individual clones in a short time and with reduced sequencing costs. Instead of whole individual clone sequencing, we sequenced the clones in a pool and the medium-large insert (7–15 kb) cloning strategy allowed us to assemble these 358 sequenced clones correctly and assign them the correct Sanger reads of clone ends. Thus, the clone-end sequences maintain the link between the living clones and the annotated contigs from 454 assemblies, and so serve to locate the clone of interest in the clone library. The retrieved data facilitate planning consecutive biochemical assays for a given clone of interest. Moreover, the choice of the easy-handling plasmid vector enables an appropriate sub-cloning strategy to be designed for gene expression in suitable vectors/hosts. This article reports the characterization of the first metagenomic clone library from the fecal sample of a healthy human volunteer.

## Materials and Methods

### Isolation of Bacterial Cells from a Fecal Sample and DNA Extraction

The study was approved by the Ethics and Research Committee of the Centre for Public Health Research (CSISP) of Valencia, Spain. The volunteer involved in this study provided a written informed consent. One ml of fresh feces from the healthy volunteer was resuspended in 3 ml of salt solution (0.9% NaCl) by vortexing and then centrifuged at 2000 rpm for 2 min. The supernatant was transferred to a 15 ml tube. Microbial cells were purified as previously described [21]. Bacterial DNA was extracted by the Ausubel protocol (1992) including lysozyme, SDS and CTAB treatment, phenol-chloroform purification and isopropanol-ammonium acetate precipitation [21], [22].

### Preparation of the Clone Library

Seven µg of extracted bacterial DNA was digested with EcoRI enzyme (Roche, ref.: 10703737001) at 37°C for 2 hours and then resolved on 0.5% TAE agarose gel at 15 V for 16 hours. DNA fragments measuring between 7 and 15 Kb were cut out from the gel without exposure to UV light. DNA elution was performed in the Elutrap device (Whatman, ref.: 10447700) running at 150 V for 3 hours. Amicon 50 K columns (Millipore, ref.: UFC503024) were used for sample concentration and for the exchange of the electrophoresis buffer into water. Fragments shorter than 1.5 Kb were completely removed to allow ligation of longer ones by adding 100 µl of Agencourt Ampure Xp magnetic beads (Beckman Coulter, ref.: 082A63881) to the DNA sample, diluted in 200 µl 10 mM Tris-HCl. DNA was bound to the magnetic beads on a magnetic particle concentrator (Invitrogen, ref.: 123-21D) and purified by 70% ethanol. Size selected DNA was finally resuspended in 20 µl 10 mM Tris-HCl.

Two µl of sample DNA was ligated to EcoRI pBluescript (Agilent, ref.: 212250) with Takara DNA ligation kit (Takara, ref.: 6024) at 16°C overnight. The ligation reaction was transformed into One Shot TOP10 electrocompetent cells (Invitrogen) at 1800 Volts (Electroporator 2510, Eppendorf). Transformed cells were incubated in 1 ml of SOC medium at 37°C for 1.5 hours and then spread on LB agar plates containing ampicillin (100 µg/ml), XGAL (50 µg/ml), and IPTG (1 mM) and incubated at 37°C overnight.

### Sequencing of Clones

White colonies were picked and placed separately in 1 ml of LB with 100 µg/ml ampicillin into 96 well plate and left to grow overnight at 37°C. Plasmid minipreps were performed using 100 µl of solution 1 (50 mM sucrose, 25 mM TrisHCl pH 8, 10 mM EDTA), 200 µl of solution 2 (0.2% NaOH, 1% SDS) and 150 µl of solution 3 (3 M potassium acetate, 2 M acetic acid, pH 4.8). Plasmid DNA was resuspended in 30 µl of water. Twenty ng of each of the 358 clones were pooled together.

The shotgun library was prepared from 1 µg of the pooled sample according to manufacturer instructions (Roche, Rapid Library Preparation Method Manual GS FLX+ Series XL+, May 2011). The sample was then sequenced on 1/8 of PicoTiterPlate by GS FLX+ system. Sequencing depth has been calculated in order to reach coverage of about 10×, distributed among the 358 clones.

Plasmid DNA obtained by miniprep from each clone (about 60 ng) was sequenced separately by the Sanger method on DNA ABI 3730 (Applied Biosystems) using M13 forward or M13 reverse primers.

### Assembly and Annotation

In order to remove vector sequences (2961 bp), Smalt 0.5.8. tool (Wellcome Trust Sanger Institute, http://www.sanger.ac.uk/resources/software/smalt/) was used and plasmid sequences coordinates were employed in the following assembly step to avoid unnecessary vector assembly. Pyrosequencing reads were assembled by MIRA3 applying typical *de-novo* genome 454 assembly parameters [23].

Aiming to a correct mapping of clone ends, vector sequences present in Sanger reads were cut out. Sanger reads were then mapped on 454 assembly by Staden package v 4.11.2 and the resulting contigs were revised manually [24].

For protein annotation, contigs longer than 1000 bp were selected. ORFs (open reading frames) were identified by Glimmer 3 [25] and annotated by KAAS - KEGG Automatic Annotation Server, KEGG BRITE [26], [27]. Annotated ORFs were further enriched by InterProScan Sequence Search [28], [29]. The InterPro database makes use of different scanning tools and integrates predictive models or signatures from diverse source repositories: BlastProDom [30], Coil [31], FPrintScan [32], Gene3D [33], HAMAP [34], HMMPanther [35], HMMPfam [36], HMMPIR [37], HMMSmart [38], HMMTigr [39], PatternScan and ProfileScan [40], Seg [28], [29], SignalPHMM [41], Superfamily [42], TMHMM [43]. InterPro combines individual strengths of these different annotation sources and provides comprehensive information about protein families, domains and functional sites. Protein names resulting from InterProScan Sequence Search were submitted to the Brenda database to obtain a general overview of possible protein application [44].

A figure with the flow chart of the proposed approach is shown in Figure S1.

### Accession Numbers

Sequences were deposited in EMBL-EBI Sequence Read Archive (SRA) with study number ERP001596 (http://www.ebi.ac.uk/ena/data/view/ERP001596).

## Results and Discussion

### Sequencing Results and Assembly

In total, 57,469 out of 87,898 reads were assembled into 473 contigs, with an average coverage of 14.67X, while N50 contig size was 8,241. The largest contig measured 23,504 bp. Twelve clones containing only the vector (false positives with no insert) were excluded from the analysis.

The hybrid assembly revealed that 57 contigs correctly matched more than one Sanger sequence, showing a probable partial digestion or that inserts can proceed from different original microbial genetic rearrangement. Only six Sanger sequences could not be mapped to 454 contigs.

The assembly results show that on using the strategy of cloning the medium-large inserts (7–15 kb) into plasmids, there is no need for additional paired-end 454 sequencing; moreover, the coverage for correct assembling was sufficient. The sequence length of the pBluescript plasmid (2961 bp) is lower than the length of commercially available fosmid of BAC vectors (8–17 kb), which reduces the number of reads containing vector sequences.

### General Overview on Annotated ORFs

Out of 473 contigs, 316 were larger than 1000 bp and used for the analysis. Glimmer3 identified 1790 ORFs. The average length of ORFs was 249 amino acids, while the shortest and the longest ones were 38 and 2381 amino acids, respectively. HMMPfam annotated 742 different proteins in our assembly and Seg scanning application identified 1,859 matches (see Table 1). The complete table of ORF annotation by all annotation tools of InterProScan Sequence Search is shown in Table S1.

**Table 1:** InterProScan annotation overview.

Annotation tool	Total number of matches	Total number of unique protein names
BlastProDom	26	18
Coil	190	1
FPrintScan	732	107
Gene3D	1312	226
HAMAP	112	95
HMMPanther	1017	165
HMMPfam	1526	742
HMMPIR	93	74
HMMSmart	318	109
HMMTigr	341	257
PatternScan	257	140
ProfileScan	384	129
Seg	1859	1
SignalPHMM	394	1
superfamily	1188	226
TMHMM	1535	1

Total number of matches and total number of unique protein names assigned by different annotation tools provided by InterProScan. This table summarizes Table S1, which contains the whole list of protein matches in our assembly. The number of matches is higher than the number of unique protein names because one type of protein could be found in several contigs or one ORF could contain several matches to the same protein.

**Figure 1:**
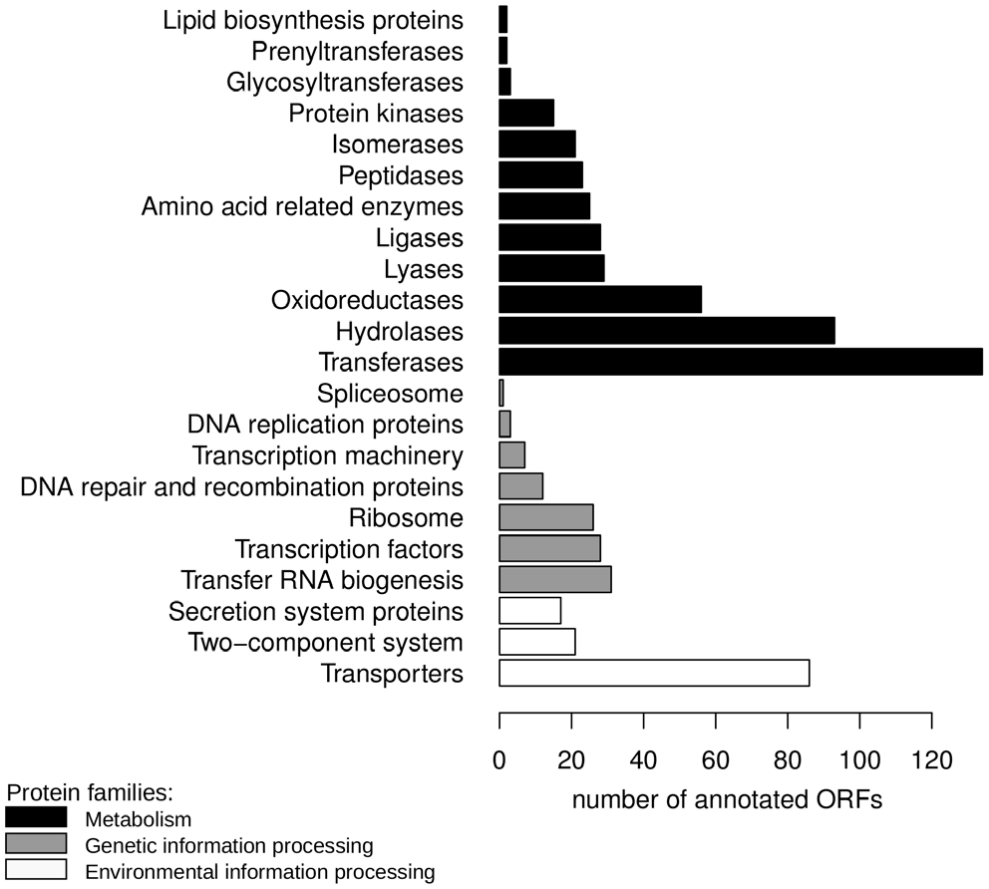
KEGG categories distribution. Distribution of KEGG categories identified among ORFs.

Figure 1 shows the distribution of KEGG annotation by protein families. The annotated enzymes corresponded to 121 different KEGG metabolic pathways. It is noteworthy that we found almost complete metabolic pathways of valine, leucine and isoleucine biosynthesis (see Figure S2).

### Annotated ORFs with Reported Industrial Applications

**Figure 2:**
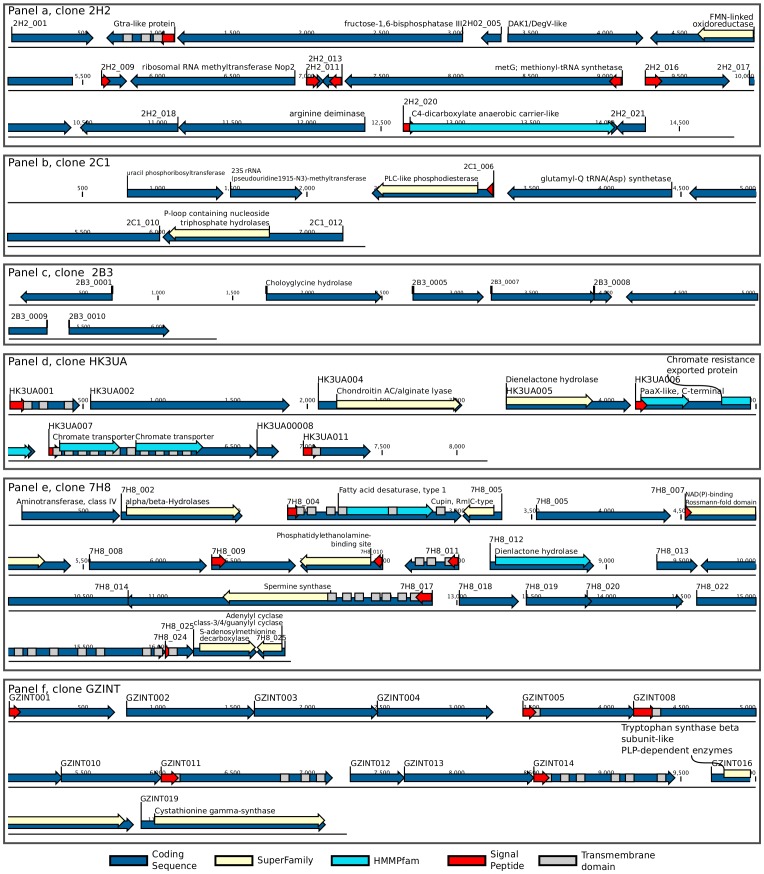
Annotated ORFs with reported industrial applications. Figure describes ORFs annotation of selected clones of interest. Annotation colors describe the kind of annotation (see legend). Every panel describes a different clone (see results section text for detailed descriptions).

The clone library derived from the fecal sample of a healthy volunteer provided genetic information of several clones containing enzymes with previously known applications. Figure 2 shows a description of some clones of interest and a summary is given in Table 2.

**Table 2:** ORFs with potential industrial or medical applications.

Protein name	Contig ID	ORF number	Contig length
Arginine deiminase	2H2	19	14.867 bp
Uracil phosphoribosyl transferase/Uridine kinase	2C1	1	7.390 bp
Choloylglycine hydrolase	2B3	4	6.393 bp
Alginate lyase	HK3UA	4	8.204 bp
Spermine synthase	7H8	17	16.889 bp
Cystathionine synthase	GZINT	16 and 19	12.265 bp

Columns describe ORF annotations, contig identifiers and ORFs identifier.

Clone 2H2 (Figure 2, panel a) contained arginine deiminase (ADI). This enzyme has also been found in bacteria, archaea (*Pseudomonas*, *Mycoplasma*, *Halobacterium*, *Lactobacillus*, *Lactococcus* [45]) and some eukaryotes, but not in mammalian cells which synthesize arginine from citrulline. Arginine auxotrophic cancer cells lack active citrulline to arginine recycling pathway and, therefore, an arginine-degrading enzyme may eradicate them effectively [46]. ADI has been tested successfully as an anti-tumoral drug for the treatment of arginine-auxotrophic tumors, hepatocellular carcinoma and melanoma [47]. ADI also improves liver function in patients with chronic hepatitis C virus (HCV) infection and selectively inhibits HCV replication *in vitro* [48]. Moreover, ADI could also be employed in the treatment of nitric oxide synthase-related neuronal diseases, which was demonstrated in a co-culture of neurons and microglia [49].

Clone 2C1 (Figure 2, panel b) contained a gene annotated as uridine kinase (uridine-cytidine kinase, HMMPanther annotation) or as uracil phosphoribosyltransferase (HMMPfam and KAAS KEGG annotations). A proposed gene therapy is based on the strategy that a non-mammalian gene encoding a certain enzyme is transduced in tumor cells and its expression catalyses the activation of a pro-drug to a cytotoxin that induces tumor cell death [50]. A cytosine deaminase-uracil phosphoribosyltransferase fusion gene has been used in clinical gene therapy trials to improve strong chemotherapeutic agents, in which 5-fluorouracil is catalyzed by cellular enzymes to fluoronucleotides, subsequently inhibiting DNA or RNA synthesis [51]–[53]. Uridine kinase and uracil phosphoribosyltransferase are enzymes catalyzing the formation of uridine 5′-monophosphate from uridine and adenine 5′-triphosphate and from uracil and phosphoribosyl-α-l-pyrophosphate in the pyrimidine salvage pathway, respectively [54]. Uracil phosphoribosyltransferase was also successfully applied in the treatment of hepatitis C virus (HCV) infection where ribavirin (1-b-D-ribofuranosyl-1, 2, 4-triazole), a synthetic nucleoside analog, is currently used in combination with interferon-α or peginterferon-α [55]. In HCV infection, because the vast majority of replication occurs in hepatocytes, selective delivery of ribavirin into those liver cells would be desirable to enhance antiviral activity and also avoid systemic side effects. In 2008, VirovicJukic showed that human uridine-kytidine kinase-1 recognizes ribavirin and phosphorylates it [56]. Introducing a phosphate group in ribavirin facilitates the preparation of a novel protein conjugate of ribavirin, which has the potential for targeted delivery to specific cell types.

Another enzyme of interest is choloylglycine hydrolase (bile salt hydrolase) found in contig 2B3 (Figure 2, panel c). Choloylglycine hydrolase is present in many bacterial species inhabiting the human gut, and has been found to have cholesterol lowering effects [57].

We found alginate lyase in contig HK3UA (Figure 2, Panel d). Alkawash (2006) demonstrated in an *in vitro* biofilm system that co-administration of antibiotics with alginate lyase from *Bacillus circulans* might benefit cystic fibrosis patients by increasing the efficacy of antibiotic in the respiratory tract [58]. Once mucoid (alginate-producing) strains of *Pseudomonas aeruginosa* have become established in the patient’s respiratory tracts, they can rarely be eliminated by antibiotic treatment alone. Alginate lyase was also found to have application in plant culture techniques *in vitro*. It has been applied successfully for the extraction of protoplasts for food research and regeneration of a variety of algal species, including brown algae, and serves as an alternative for various mechanical and chemical methods [59].

### Clones Related to Potential Applications Treating Human Enzyme Deficiencies

Several studies indicate an association between common neuro-developmental disorders and gut microbiota. The microbial colonization process triggers signaling mechanisms that can influence central nervous system development and might be linked to autism [60], [61]. In the clone library, we found enzymes with a homolog in humans, whose deficiency has been described to lead to neurological diseases. These clones should be investigated in greater depth to explain the interactions between gut microbiota and the human central nervous system.

It is known that bacteria can mediate gene transfer, which has led to the utilization of various bacterial strains in gene therapy [62]–[65]. Several publications demonstrate the considerable potential of using genetically modified lactic acid bacteria to deliver therapeutic peptides and proteins to the mucosa [66]. Greater knowledge of the interactions between humans and their gut bacteria may open up new hypothetical therapeutic approaches based on gene therapy for neurological diseases. For example, we found an ORF in contig 7H8 (Figure 2, panel e) annotated as spermine synthase (HMMPfam) or spermidine synthase (KAAS KEGG). Spermidine synthase converts putrescine into spermidine, and spermine synthase converts spermidine into spermine [67]. Spermine deficiency in human causes the Snyder-Robinson syndrome, an X-linked mental retardation disorder [68]. Wang (2004) suggested that attempts to increase spermine by dietary manipulation, drug treatment or gene therapy may be successful in preventing the Snyder-Robinson syndrome [69].

Cystathionine β-synthase (CBS) is a vitamin B6-dependent trans-sulfuration enzyme needed to synthesize cysteine from methionine. A CBS deficiency causes homocystinuria, a rare autosomal recessive disease, characterized by mental retardation, psychiatric disturbances, skeletal abnormalities, and vascular disorders [70]. Only around half of the patients with CBS deficiency respond to pyridoxine therapy [71]; thus, gene therapy might be an alternative for those that do not respond to this treatment. Oh and collaborators (2004) used three kinds of human CBS cDNA to construct vectors for gene therapy, demonstrating positive effects on homocystinuria affected mice [72]. In our clone library, the ORF 16 of contig GZINT was annotated as cystathionine β-synthase and ORF 19 as cystathionine γ-synthase (Figure 2, panel f).

### Concluding Remarks

Metagenomics of the human microbiome can provide genetic information about the DNA of bacteria inhabiting human-related ecological niches, adapted to physiological conditions such as temperature, pH, redox potential, etc. The assembly and annotation of clones produced from a library of this kind reveal the presence of several proteins for which industrial or medical applications have already been reported. They can also be used to study proteins of unknown function. The screening of a clone library with inserts of 7–15 Kb by 454 pyrosequencing enabled us to obtain the annotation information of the genes present in each clone without the need of prior and expensive *ad hoc* biochemical screening. The approach used in this paper started by analyzing 358 clones and ended up with 316 assembled and annotated contigs. The proposed method is perfectly scalable enabling work to tackle larger clone libraries, proportionally increasing sequencing efforts. This strategy enables the link to be maintained between the information and the living clones, providing annotation of the whole library; thus, particular clones of interest can undergo further testing by the most appropriate biochemical assay and/or sub-cloning for appropriate selection of vectors and/or host, if required.

## Supporting Information

Figure S1 **Protocol flow chart.** The figure summarizes the protocols applied to construct the clone library, pyrosequencing of pooled clones, individual clone Sanger end sequencing, contigs/clone-ends matching and annotations.
(TIFF)

Figure S2 **Valine biosynthesis pathway.** Green frames indicate enzymes found in the library.
(TIFF)

Table S1 **Whole library InterPro annotation table.** Columns describe: ORF name bounded to the original clone name in the library; amino acid length; InterPro inquired database; database match; match description; ORF match start position; ORF match end position; match p-value; date of search; InterPro match code; protein name; protein description.
(ZIP)
