# Supplementary figures and images for: Hybrid Sequencing Approach Applied to Human Fecal Metagenomic Clone Libraries Revealed Clones with Potential Biotechnological Applications
Source: PLoS One. 2012 Oct 17;7(10):e47654. doi: 10.1371/journal.pone.0047654 (PMC3474745; doi:10.1371/journal.pone.0047654)

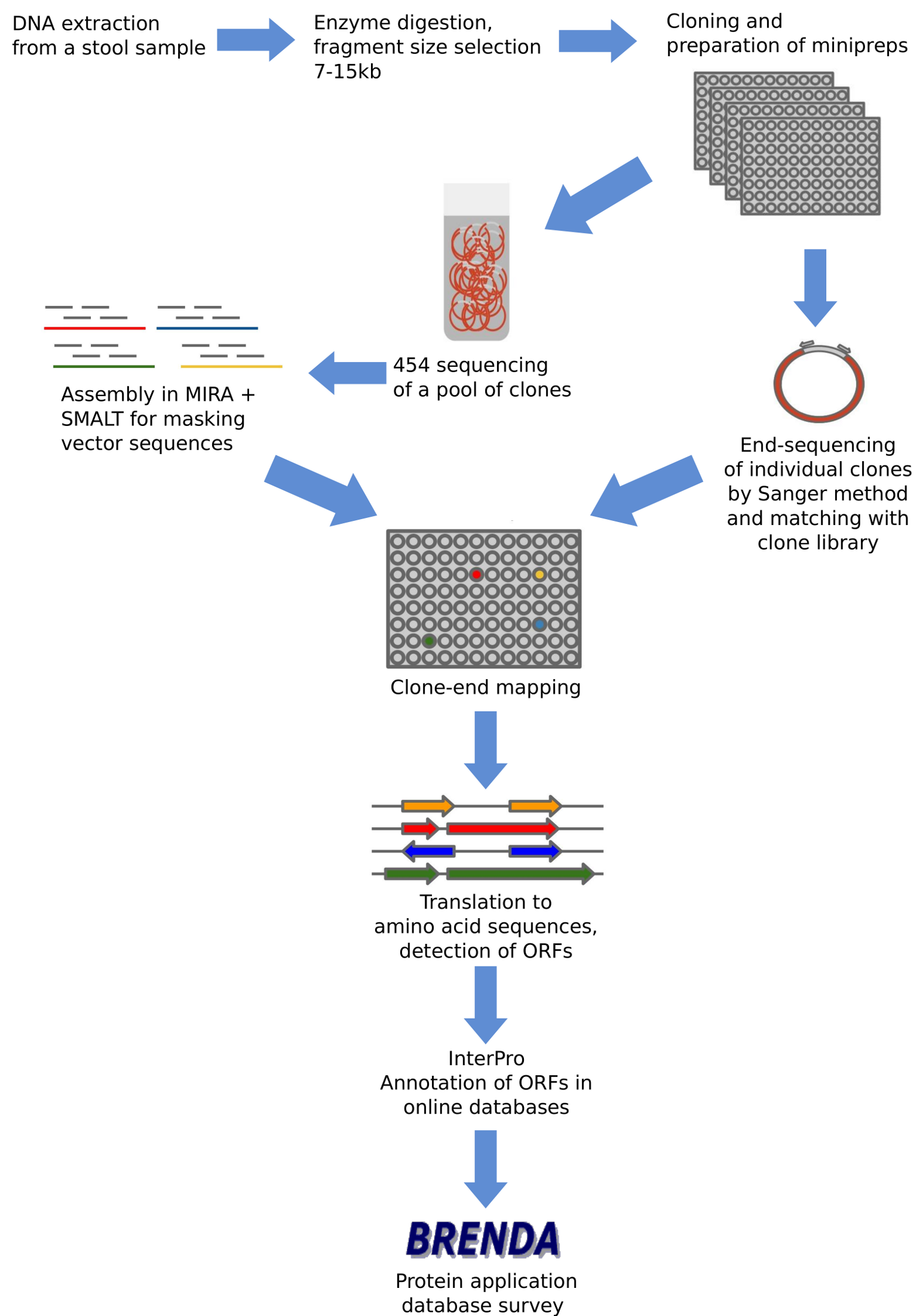

Supplement: Figure S1 — Protocol flow chart. The figure summarizes the protocols applied to construct the clone library, pyrosequencing of pooled clones, individual clone Sanger end sequencing, contigs/clone-ends matching and annotations. (TIFF) [file pone.0047654.s001.tiff]

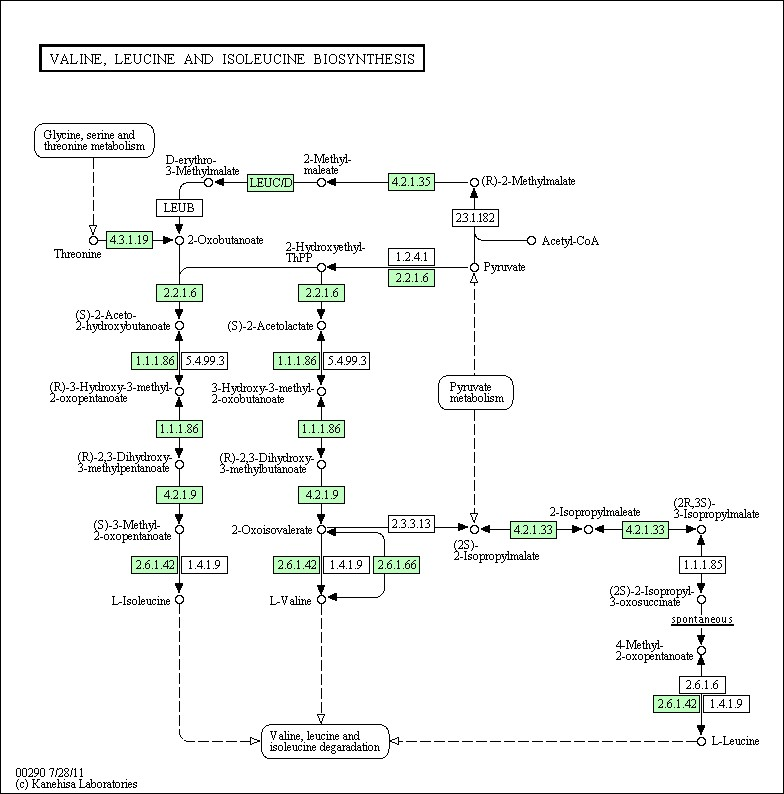

Supplement: Figure S2 — Valine biosynthesis pathway. Green frames indicate enzymes found in the library. (TIFF) [file pone.0047654.s002.tiff]
